# Qualitative and quantitative: the yin and the yang or the light and the dark sides of medical education?

**DOI:** 10.1007/s40037-015-0162-3

**Published:** 2015-01-27

**Authors:** Robert K McKinley

**Affiliations:** Keele University School of Medicine, Keele University, Keele, ST5 5BG UK

Research and scholarship in medical education have had a poor press, perhaps deservedly so. Our work had been criticized as too often atheoretical, [1] methodologically weak, [1–4] irrelevant [4] and inaccessible to educators at the coal face [2]. It is too often underfunded, [1, 2, 5, 6] and we have been described as institutionally and professionally insular [1, 4]. In many ways our response to these charges has been admirable. We have had clearly articulated calls to enhance the quality of our outputs from leaders in the field, [7] trenchant debate [3, 8–10] and measured contributions as to exactly what ‘quality’ in research into medical education might be [11, 12].

And what advice do we, the older or more experienced members of the medical education community, now give those who seek to replace us? Or perhaps, we should ask what do they hear? I believe, to paraphrase the adage from the 1850s ‘Go west young man, go west and grow up with the country’, that what they hear is ‘Go qualitative new colleague, go qualitative!’ Like the advice given in ninteenth century North America, this has much to commend it: we have wide prairies to explore and understand and populate with ideas and room in which to make a reputation. And this is happening. A search of MEDLINE shows that, since 2000, the number of papers with ‘qualitative’ but not ‘quantitative’ in their title and abstract in Medical Education, Medical Teacher, BMC Medical Education and Academic Medicine outnumbered whose with the obverse (‘quantitative’ and not ‘qualitative’ in the title and abstract) by 6.5–1. The research of many of our superstars is strongly (albeit not exclusively) qualitative. Of the last five winners of the ASME young researcher awards, four have been qualitative or strongly qualitative in nature, the other was action research with a strong qualitative component.

I worry, however, that the pendulum has swung too far and others may share aspects of my concerns [13]. Yes, we are building theory but the value of a good theory is that it enables predictions which can be tested. Some of that testing requires measurement. We need research which seeks to quantify whether a theory-based intervention has the effects which were predicted and whether that difference is worth the candle. We need to know whether our theory building actually makes differences which matter to educators on the ground, to our learners who have entrusted us with their future, and to their patients in the future. As Yogi Berra may have said, ‘In theory there is no difference between theory and practice. In practice there is.’

The current predominance of qualitative research in medical education contrasts with the discourse in medical research though the 1990s. Then, those who championed qualitative research were working to establish the credibility of their chosen paradigm and to shift the perception that their work was unscientific and lacking in rigour [14, 15]. They were riding a wave: influential guidelines were soon published [16, 17] and since updated [18]. Now mixed methods research combining qualitative and quantitative approaches is commonplace in health technology assessment and health services research and few research programmes can expect funding unless they bring the gaze of both to the problem being examined.

Quantitative research in medical education is important and will continue to be important. The descriptive work of Papadakis [19] and Tamblyn [20−22] has been hugely influential. These enormously painstaking pieces of ground breaking work hint at the potential that lies within the enormous datasets being assembled by our governments, administrators and regulators. These datasets are ripe for the application of ‘big data’ research techniques. Such approaches are being used to inform clinical care and we need to learn to use them to answer questions in education and training. For example, national licensing examination scores are associated with indices of care provided by doctors [22]. Can we identify doctors at increased risk of error and intervene to reduce harm to patients? This will be difficult: we will need to develop new skills to link datasets and validate data, to master new study designs and to understand how our results may be confounded and whether we can account for it.

We also need to find out whether aspects of our curricula make a difference. This will need multi-institutional research which will require building trust, identifying worthwhile questions which we can answer and finding outcome measures which, although imperfect, will provide useful information. For example does the Conscientiousness Index [23] have cross-institutional validity and predict graduates at higher risk of suboptimal practice? Is the answer important? Should an affirmative answer change educational practice? I would argue ‘Yes it is’ and ‘It must’. Are we developing the people who can answer questions such as these? I fear we are not.

If we have the courage to explore these tough questions, we may find answers that are unexpected or even distressing. But these answers are the gold dust of science; to understand them fully, we will need mixed methods research that blends the strengths of both traditions.

Goldszmidt et al., [24] in their reply to the letter from Van Merrienboer, [13] argue that the dichotomy between objectivity and subjectivity is false. I agree that these words are often not useful, but my argument is subtly different: research which seeks to explain and understand should not be separated from that which enumerates and describes. They are complementary and will provide better answers when used together.

Has the pendulum swung too far? Perhaps it has; at the very least, it is time to rethink the balance between quantitative and qualitative inquiry. One is not the dark and the other the light side but rather the yin and the yang: one without the other is insufficient. We need to learn from the health services research and health technology assessment communities and invest in the skills required for both.
